# Extracellular secretion of Carocin S1 in *Pectobacterium carotovorum *subsp. *carotovorum *occurs via the type III secretion system integral to the bacterial flagellum

**DOI:** 10.1186/1471-2180-9-181

**Published:** 2009-08-27

**Authors:** Yung-chieh Chan, Huang-Pin Wu, Duen-yau Chuang

**Affiliations:** 1 Department of Chemistry, National Chung-Hsing University, 250 Kuo Kuang Road, Taichung, Taiwan, Republic of China; 2 Division of Pulmonary Medicine, Department of Internal Medicine, Chang Gung Memorial Hospital, Keelung, Taiwan, Republic of China

## Abstract

**Background:**

*Pectobacterium carotovorum *subsp. *carotovorum *is a phytopathogenic enterobacterium responsible for soft rot, a disease characterized by extensive maceration of the affected plant tissue. This species also produces two or more antibacterial substances called bacteriocins, which enhance its competitiveness against related rival species. However, the secretion mechanism for low-molecular-weight bacteriocin is still unknown.

**Results:**

A mutant (*flhC*::Tn*5*) that did not secrete the low-molecular-weight bacteriocin (LMWB), Carocin S1, was generated by Tn*5 *insertional mutagenesis. Sequence analysis indicated that this insertion disrupted open reading frame 2 (ORF2) and ORF3 of this strain. Deletion and rescue experiments indicated that ORF2 and ORF3 were both required for extracellular LMWB secretion. The ORF2 and ORF3 sequences showed high homology with the *flhD *and *flhC *gene sequences of *Pectobacterium carotovorum *subsp. *atroseptica*, *Serratia marcescens, Yersinia enterocolitica*, and *Escherichia coli*, indicating that they likely encoded key regulatory components of the type III flagella secretion system.

**Conclusion:**

Thus, the extracellular export of Carocin S1 by *Pectobacterium carotovorum *subsp. *carotovorum *appears to utilize the type III secretion system integral to bacterial flagella.

## Background

*Pectobacterium carotovorum *subsp. *carotovorum *is a phytopathogenic enterobacterium responsible for soft rot, a disease characterized by extensive plant tissue maceration caused by a variety of secreted enzymes. The major pathogenicity determinants are an arsenal of extracellular pectinases, including several pectate lyase isozymes: pectin lyase, pectin methylesterase, and pectin polygalacturonase. In addition, a range of other degradative enzymes, such as cellulase and proteases, play equivocal roles in virulence [1]. *Pectobacterium carotovorum *subsp. *carotovorum *also produces one or more antibacterial substances called bacteriocins, which enhance their competitiveness with other related rival species [2]. The ability of this bacterial species to produce bacteriocin has been exploited in many biological control programs for the soft-rot disease of Chinese cabbage [3-5]. In view of this, identification and cloning of the gene(s) controlling bacteriocin production may facilitate the development of wider and more innovative control methods, such as the cloning of these gene(s) into Chinese cabbage, tobacco, and other susceptible plants to produce resistant cultivars.

In our previous paper, the *brg *gene was found to encode a regulator required for the expression of the low-molecular-weight bacteriocin (LMWB) in a strain of *Pectobacterium carotovorum *subsp. *carotovorum *[1]. The gene is homologous to *hfq *and encodes a protein with similar functions [1,6]. The genetic determinant encoding LMWB synthesis was designated the *Carocin S1 *genetic determinant, which consists of two structural genes, *caroS1K *(encoding killer protein) and *caroS1I *(immunity protein). Clear zones of inhibition around CaroS1K producer colonies are due to CaroS1K antibiotic activity. Carocin S1-associated nuclease activity has also been demonstrated [7]. The carocin S1 gene has been isolated from *Pectobacterium carotovorum *subsp. *carotovorum *89-H-4 and functionally expressed after introduction into *Pectobacterium carotovorum *subsp. *carotovorum *Ea1068a (a non-bacteriocin-producing strain). From our previous studies, glucose, as well as SOS agents, can also induce the *carocin S1 *gene.

Using the same Carocin S1-producing strain of *Pectobacterium carotovorum *subsp. *carotovorum*, genes controlling the LMWB have been cloned and sequenced, and homology to the *flhD/C *operon demonstrated. The *flhD/C *operon is a regulator that activates expression of genes required for flagella assembly. However, its activity depends on environmental stimuli (e.g., cyclic AMP levels, temperature, heat shock, osmolarity, membrane biosynthesis, and H-NS protein [8]), cell division, flagella formation, and motility [9-11].

A number of Gram-negative pathogenic bacteria have evolved a specialized type III protein secretion system to deliver effector virulence proteins into host cells [12,13]. There are two types of type III secretion systems: the translocation-associated type III secretion system (T3aSS) and the bacterial flagellum type III secretion system (T3bSS). The various bacterial type III secretion systems characterized thus far all have Sec independence, ATPase dependence, presence of a hollow filamentous organelle that extends from the outer membrane, a cell-envelope-spanning secretion channel, and nine conserved proteins [14].

The bacterial flagellum type III secretion system also serves as the bacterial flagellum (a biological nanomachine with an ion-powered rotary motor). For the flagellum, the T3bSS apparatus functions to secrete components including the rod, hook, and filament subunits for extracellular assembly. The core of the flagellum is hollow, and secreted subunits polymerize at the growing end of the flagellum. A cap at the tip of the flagellum ensures efficient polymerization of secreted subunit proteins [15,16]. This secretion apparatus is just one mechanism utilized by Gram-negative plant and animal pathogens for the secretion and translocation of virulence determinants into susceptible eukaryotic cells [17]. In *Salmonella typhimurium*, the expression of class 1 genes (i.e., *flhD *and *flhC*) activates expression of genes required for flagella assembly and regulates expression class 2 genes (e.g., *fliAZY *and *flhBAE*), which in turn regulates expression of class 3 genes encoding flagellar structural proteins (e.g., *fliC*, *flgMN*, and *MotAB*) [18]. In *Xenorhabdus nematophila*, it was shown that the EnvZ-OmpR-FlhDC-FliA regulatory network coordinately controls flagella synthesis as well as exoenzyme and antibiotic production [8].

In this paper, we describe the transcriptional regulation of *fliC *and *flhA *expression by *flhD/C *and also show that *flhD/C *has an effect on extracellular secretion of the Carocin S1 protein, but not on Carocin S1 gene expression. Our results indicate that the type III secretion system of *Pectobacterium carotovorum *subsp. *carotovorum *has a new secretory function.

## Methods

### Bacterial strains, plasmids, media, and growth conditions

The strains and plasmids used are shown in Table 1. *Pectobacterium carotovorum *subsp. *carotovorum *strains were propagated at 28°C in 1.4% nutrient agar (NA) or with shaking in Luria-Bertani (LB) medium with NaCl (5 g/L). *E. coli *strains were propagated at 37°C in LB medium with shaking. Rifampicin, kanamycin, and ampicillin (all at 50 mg/L) were added to either medium when necessary.

**Table 1:** Bacteria and plasmids used in this study

Bacterium or plasmid	Relevant characteristics	Source
*E. coli*		
1830	*pro*^-^*met*^-^*Km*^r^*Nm*^r^, containing transposon Tn*5 *on the ?sucidal? plasmid pJB4JI	Gantotti et al. [37]
DH5	*supE44hsdR17recA1endA1gyrA1thi-1relA1*	Hanahan and Reusch et al [26,38]
		
*Pectobacterium carotovorum *subsp. *carotovorum*		
89-H-4	putative biocontrol agent	Laboratory stock
H-rif-8-6	89-H-4, *Rif*^r^	this work
Ea1068	wild type	Laboratory stock
T-29	wild type	Laboratory stock
E108	wild type	Laboratory stock
A-100	wild type	Laboratory stock
86-H-2	wild type	Laboratory stock
TH12-2	H-rif-8-2, *flhC:: *Tn*5, Rif*^r^, *Kan*^r^	this work
KH17	H-rif-8-2, *flh D::Kan, Rif*^r^, *Kan*^r^	this work
FliC-KO	H-rif-8-2, *fli C::Kam, Rif*^r^, *Kam*^r^	this work
FlhA-KO	H-rif-8-2, *flh A::Kam, Rif*^r^, *Kam*^r^	this work
		
plasmid		
pBR322	*Amp*^r^, *Kan*^r^	Bolivar et al [39]
pBYL2DC	*Amp*^r^, *flhDC*	this work
pBYL2C	*Amp*^r^, *flhC*	this work
pBYL2D	*Amp*^r^, *flhD*	this work
pBFC	*Amp*^r^, *fliC*	this work
pBFA	*Amp*^r^, *flhA*	this work

*Amp*^r ^indicates ampcillin resistance, *Rif*^r ^indicates rifampicin resistance, and *Kan*^r ^indicates Kanamycin resistance.

### Bacterial mating

Bacterial mating was carried out on NA using the membrane-filter mating method [14] with 0.22-μm pore size membrane filters (Millipore, Inc. Bedford, MA). The filters were placed on NA and incubated overnight at 28°C. Appropriate dilutions of each progeny suspension were spread on modified Drigalski's agar plates [19] containing 50 μg/ml rifampicin and kanamycin and incubated at 28°C for 24–48 h before the colonies were isolated.

### Bacteriocin assays

Bacteriocin production was examined as described previously [20] in hard IFO-802 (with 1.4% agar) and soft IFO-802 (with 0.65% agar) medium. Growth inhibition zones around the colonies were considered as an indication of bacteriocin production.

### Genetic engineering techniques

Previously described techniques were used to isolate the plasmids of *Pectobacterium carotovorum *subsp. *carotovorum *[21,22] and *E. coli *[23]. Total DNA was isolated as previously described [22]. Oligonucleotide DNA primers were synthesized by MDE Bio Inc. (Taipei, Taiwan). Reagents were purchased from Takara Co. (Tokyo, Japan). Previously detailed protocols were utilized for the general polymerase chain reaction (PCR) [24] and thermal asymmetric interlaced PCR (TAIL-PCR) [25], except that in the latter technique the annealing temperature of specific primers was decreased from 63°C to 60°C. For TAIL-PCR, specific primers complementary to the respective sequences of Tn*5 *(PR-1, PR-2, PR-3, PF-1, PF-2, and PF-3) or known sequences after the first TAIL-PCR analysis (TH12-2F1, TH12-2F2, TH12-2R1, and TH12-2R2) were synthesized (Table 2). In addition, three arbitrary degenerate primers designated N-1, N-2, and N-3 were used (Table 2).

**Table 2:** Primers used in this study^a^

Primer		Sequence (5'→3')
PR-1	.........	5'-GCCGAAGAGAACACAGATTTAGCCCA
PR-2	.........	5'-CCGCACGATGAAGAGCAGAAGTT
PR-3	.........	5'-CAGATCTCTGGAAAACGGGAAAGG
PF-1	.........	5'-AGAGAACACAGATTTAGCCCAGTCGG
PF-2	.........	5'-CCGCACGATGAAGAGCAGAAGTTAT
PF-3	.........	5'-GATCCTGGAAAACGGGAAAGGTTC
TH12-2F1	.........	5'-GATGGTGAAATTGGCAGAAAC
TH12-2F2	.........	5'-GGACATTAGTCCGGTTTGTTG
TH12-2R1	.........	5'-CAACAAACCGGACTAATGTCC
TH12-2R2	.........	5'-GTTTCTGCCAATTTCACCATC
N-1	.........	5'-NGTCGA(G/C)(A/T)GANA(A/T)GAA
N-2	.........	5'-GTNCGA(C/G)(A/T)CANA(A/T)GTT
N-3	.........	5'-(A/T)GTGNAG(A/T)ANCANAGA
P-3	.........	5'-CTCGACGTTGTCACTGAAGCGGGAAG
P-4	.........	5'-AAAGCACGAGGAAGCGGTCAGCCCAT
DY-SR1	.........	5'-GAAATCGATCACCGCCTTCACAC
DY-SF1	.........	5'-AAAGAATTCTTCAGTCGCGTTG
flhA-sen	.........	5'-TCACTCAACGTTGCATCTAC
flhA-anti	.........	5'-CAAGATGTTGGCCAACAGATG
fliC-sen	.........	5'-TCGGTGCGAATGATGGTG
fliC-anti	.........	5'-AACGCAGCAGTGACAGC
fliC-Fu-sen	.........	5'-TGGTTTTATCCACGACTCAC
fliC-Fu-anti	.........	5'-ATGCAGCAGGATCCAGAAC
flhA-Fu-sen	.........	5'-TCACTCAAGCTTGCATCTAC
flhA-Fu-anti	.........	5'-CGGATTGTCGACTAGCTGG

^a ^All primers were purchased from MDE Bio Inc., Taipei, Taiwan

TAIL-PCR products were sequenced using an ABI PRISM Dye Terminator Cycle Sequencing Ready Reaction kit (Applied Biosystems, Foster City, CA). Cycle sequencing was carried out in a GeneAmp System 9600 thermocycler (Applied Biosystems). Sequencing was carried out according to the manufacturer's protocol using an ABI 373S automated DNA sequencer 373S (Applied Biosystems).

Southern and colony hybridizations, probe labeling, and detection were performed by using a DIG DNA Labeling and Detection kit (Boehringer Mannheim GmbH, Mannheim, Germany) as described by the manufacturer. Hybridization was performed overnight, and the membrane was washed according to the recommendations of the manufacturer.

DNA electrophoresis, restriction digest, ligation, and transformation procedures for *E. coli *were performed as previously described [24]. Plasmid DNA transformation for *Pectobacterium carotovorum *subsp. *carotovorum *was performed using two previously described methods [26,27] following an incubation at 35°C until the optical density (550 nm) of the culture was 0.40 to 0.55.

### Subcloning of *flhD/C *DNA from H-rif-8-6

The DNA fragment of *flhD/C *was amplified by PCR from H-rif-8-6 using oligonucleotide primers DY-SF1 and DY-SR1. The *flhD/C *DNA containing product was digested with restriction enzymes *Cla*I and *Eco*RI and subcloned into plasmid pBR322. The new plasmid was designated pBYL2DC. One hundred transformed colonies were isolated using selective LB agar containing 100 μg/ml of ampicillin after the transfer of pBYL2DC into *E. coli *DH05. The presence of the *flhD/C *DNA was detected by colony hybridization using *flhD/C *DNA probes and electrophoresis after digestion with *Cla*I and *Eco*RI to yield the expected 1.3-Kb DNA fragment bearing *flhD/C*. The pBYL2DC DNA was isolated from DH05/pBYL2DC and transferred into the insertion mutants of *Pectobacterium carotovorum *subsp. *carotovorum *TH12-2. One hundred colonies were isolated by selection on modified Drigalski's medium containing 50 μg/ml of kanamycin, rifampicin, and ampicillin. The *flhD/C *DNA was detected as previously described.

### Construction of the null alleles of *flhD*, *fliC*, and *flhA *genes

The *flhD *gene was isolated from pBYL2DC by digesting with *Bsm*I, which cleaves at two sites in pBYL2DC and thereby conveniently deletes *flhC *from the operon. The resulting plasmid was designated pBYL2D. A kanamycin resistant gene from pACYC177 was isolated, made blunt-ended using a DNA-blunting kit (Takara Co., Tokyo, Japan), and inserted in the unique *Eco*RV site of the *flh*D gene. The resulting plasmid was designated pBYL2D-Kan. The pBYL2D-Kan was re-isolated and linearized after *Hpa*I and *Ssp*I restriction enzyme digestion, which deleted the ampicillin resistance gene and replication site of the plasmid. The linearized construct was transferred into H-rif-8-6, resulting in the homologous replacement of the native *flhD *gene and generating a null allele.

The DNA fragment of *fliC *was amplified by PCR from H-rif-8-6. After PCR amplification using two oligonucleotide primers (fliC-sen and fliC-anti), the partial *fliC *DNA fragment was purified, digested using *Ah*dI and *Hin*dIII, and subcloned into plasmid pBR322 to generate the *fliC *plasmid. A kanamycin resistant gene from pACYC177 was isolated, made blunt-ended, and inserted into the unique *Sal*I site of the *fliC *gene. The resulting plasmid was designated pfliC-Kan. The pfliC-Kan was linearized after *Ah*dI and *Hin*dIII restriction enzyme digestion, which deleted the ampicillin resistance gene and replication site of the plasmid. The linearized construct was transferred into H-rif-8-6 resulting in the homologous replacement of the native *fliC *gene and generating a null allele.

The DNA fragment of *flhA *was amplified by PCR from H-rif-8-6 using oligonucleotide primers flhA-sen and flhA-anti. The partial *flhA *DNA fragment was purified, digested using the restriction enzymes *Cla*I and *Eco*RI, and subcloned into plasmid pBR322 using T4 ligase to generate the *flhA *plasmid. A kanamycin resistant gene from pACYC177 was isolated, made blunt-ended, and inserted in the unique *Sal*I site of the *flhA *gene. The resulting plasmid was designated pflhA-Kan.

### Computer analysis of sequence data

The nucleotide sequence and the deduced amino-acid sequence of FlhD/C were compared using the BLAST and FASTA programs of the National Center for Biotechnology Information server. Sequence data were compiled by DNASIS-Mac software (Hitachi, Tokyo, Japan).

### RNA preparation and Northern hybridization

Bacteriocin synthesis medium (BSM; 0.5% sucrose, 0.1% NH_4_Cl, 0.2% KH_2_PO_4_, and 0.02% MgSO_4_·7H_2_O [pH = 7.5]) was used to produce Carocin S1. Total RNA was extracted from cells (*Pectobacterium carotovorum *subsp. *carotovorum *harboring constructs) that were grown without drugs at 28°C. To determine the stability of H-rif-8-6, TH12-2, TH12-2/pBYL2C, KH17, and KH17/pBYL2D strains, culture samples (8 ml each; with rifampicin [0.2 mg/ml] added when cell density was ~150 Klett units to block bacterial contamination) were withdrawn at various time points and transferred to tubes containing 5 mL of ice-cold water. Total RNA was extracted using Trizol (Invitrogen, Carlsbad, CA) according to the manufacturer's protocol.

Northern blot hybridizations were performed using 10 μg of total RNA. RNA samples were denatured in RNA sample buffer at 65°C for 10 min. The buffer consisted of 250 μL formamide, 83 μL of 37% (w/v) formaldehyde, 83 μL of 6× loading dye (Promega, Madison, WI), 50 μL of 10× morpholinepropanesulfonic acid (MOPS; 20 mM MOPS and 5 mM sodium acetate) buffer, 1 mM EDTA (pH 7.0), and 34 μL of distilled water. RNA samples were separated on 1% agarose gels containing MOPS buffer with 2% (v/v) formaldehyde. DNA probes were synthesized by PCR using specific oligonucleotides (template sequences): PCAR-R3 (for *caroS1K*), PflhC-R1 (for *flhC*), and PflhD (for *flhD*) derived from *Pectobacterium carotovorum *subsp. *carotovorum *(Table 2). Template DNAs (*caroS1K*, *flhD*, and *flhC*) were obtained by PCR amplification. The probes were nonradioactively labeled by random priming using a digoxigenin (DIG) High Prime kit (Roche, Basel, Switzerland). To add the correct amount of probe for hybridization, a serial dilution of each probe (0.05–10 pg) was spotted on a nylon membrane, and the labeling sensitivity (amount of labeled DNA per spot) was determined. RNA was transferred overnight to a positively charged nylon membrane (Amersham Biosciences, Buckinghamshire, England) by capillary transfer using 20× SSC (0.3 M NaCl and 0.03 M sodium citrate, pH 7.0). The membrane after hybridization (performed for 16 h at 50°C in DIG Eazy Hyb buffer solution; Roche) was washed, and the specific transcripts on the blots were detected using a DIG luminescence detection kit (Roche) according to the manufacturer's protocol.

### Motility test

A sterile loopful of bacterial cells was carefully inoculated vertically into tubes containing soft agar (IFO-802 medium with 0.5% agarose). After incubation for one month, motility was determined by migration and/or outgrowth of bacterial cells from the original inoculation line.

## Results

### Isolation of transposon insertion mutants

Conjugation of strain H-rif-8-6 with *E. coli *1830 led to the isolation of 3000 colonies that grew on the selective plates containing 50 μg/mL rifampicin and kanamycin. Their antibiotic resistance was ascertained by rechecking growth on the selective medium and was found to be a stable property.

### Bacteriocin assay of Tn*5 *insertional mutants

**Figure 1:**
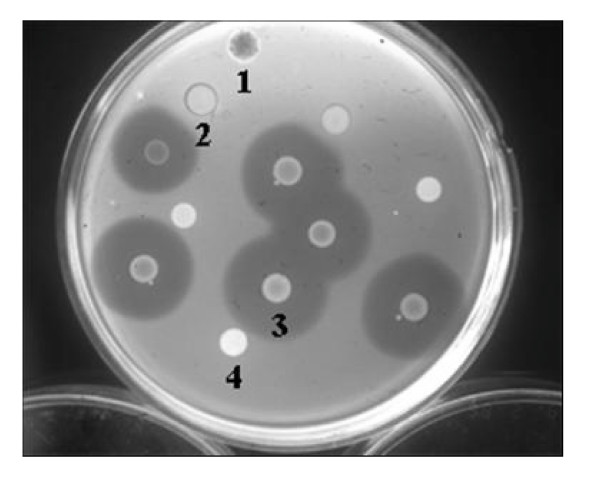
**Bacteriocin activity of Tn*5 *insertion mutants of the *Pectobacterium carotovorum *subsp. *carotovorum *strains**: 1, *Serratia *sp. (marker); 2, TH12-2 (Tn*5 *insertion mutant, *flhC*::Tn*5*); 3, H-rif-8-6 (parent); 4, *E. coli *1830/pJB4JI (containing Tn*5*). The unlabeled strains are all Tn*5 *insertion mutants of the H-rif-8-6 parental strain. Strain Ea1068 was used as an indicator for bacteriocin activity.

The bacteriocin activity of the putative insertion mutants was examined. The diameters of the inhibition zone typical were smaller around the putative mutant strains than parental strains, indicating the possibility that a gene related to Carocin S1 production had been inserted into the Tn*5 *transposon (Fig. 1).

### Detection of Tn*5 *insertions in the mutants

To ascertain whether a Tn*5 *insertion had actually occurred in the putative mutant strains, nested-PCR was used to amplify the *nptII *gene [28] using the oligonucleotide primers P-3 and P-4 (Table 2). A total of 97% of the test isolates but not H-rif-8-6 produced a 500-bp DNA fragment that did not harbor the Tn*5 *insertion. Southern blot hybridization confirmed these results (data not shown).

### Amplification of the DNA at the Tn*5 *insertion junction site and sequence analysis

**Figure 2:**
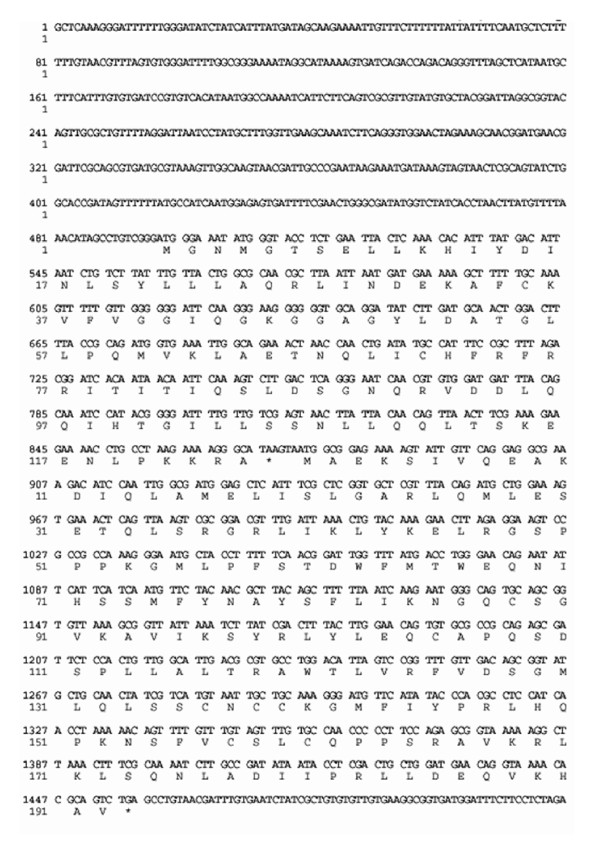
**Nucleotide sequence of the *flhD *and *flhC *genes with the deduced amino-acid sequence of their respective proteins (FlhD and FlhC)**. The nucleotide sequence of fragments (positions 497-68 and 875-1453) represent *flhD *and *flhC *genes, respectively.

TAIL-PCR was used to analyze the DNA sequences at the junctions of the Tn*5 *insertions. After the first TAIL-PCR experiment, two or more differently sized DNA fragments were obtained from each sample. All fragments were isolated by electrophoresis, purified, and sequenced and corresponding DNA fragments were shown to have the same sequence. Based on the sequence obtained from the first TAIL-PCR experiment, specific primers (TH12-2F1, TH12-2F2, TH12-2R1, and TH12-2R2) were synthesized for a second TAIL-PCR experiment. Subsequently, a nucleotide sequence of 1963 base pairs was obtained. The direction of transcription determined by analysis of the Tn*5 *insertions showed that two complete open reading frames (ORF2 and ORF3) were present and that Tn*5 *was located in ORF3 between base pairs 1312 and 1313. The 3' end of another open reading frame, ORF1, was located upstream of ORF2, and the 5' end of ORF4 was located downstream from ORF3 (Fig. 2).

### Homology with other genes and proteins

The predicted amino-acid sequences of ORF2 and ORF3 were compared to other known genes using the Swiss-Prot protein sequence data bank. A significant similarity was found between ORF2 and ORF3 of *Pectobacterium carotovorum *subsp. *carotovorum *and the *flhD *and *flhC *genes, respectively, of *Pectobacterium carotovorum *subsp. *atroseptica *(95% similarity), *Serratia marcescens *(86% similarity), *Yersinia enterocolitica *(84% similarity), and *E. coli *(80% similarity). Thus, ORF2 was designated as *flhD*, and ORF3 as *flhC*.

### Bacteriocin expression, isolation, and activity assay

**Figure 3:**
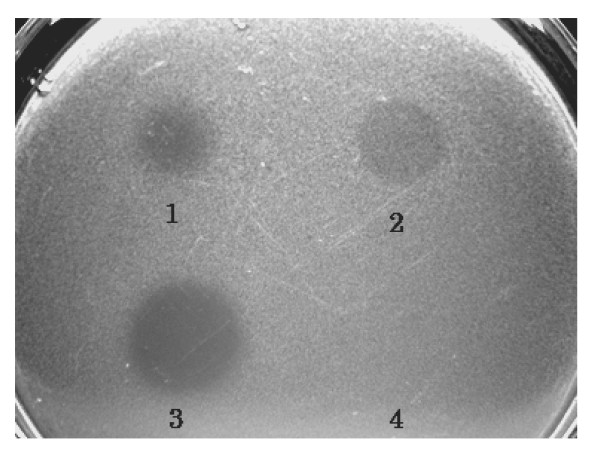
**Analysis of the killing activity of purified Carocin S1**. Intracellular solution was isolated from Hi-rif-8-6 (1) and TH12-2 (3) strains. Extracellular solutions from Hi-rif-8-6 (2) and TH12-2 (4) strains were assayed for killing activity by addition to indicator plates containing strain Ea1068.

Bacteria in BSM medium were incubated in a sterilized stainless steel box with a stainless steel cover at 28°C for 24 h without any light. After centrifugation, the extracellular solution and cells were separated and collected. The cells were homogenized by sonication, and ammonium sulfate was added to 80% saturation to precipitate the protein. The precipitate was collected on a 0.45-μm cellulose filter. One milligram of precipitated protein was dissolved in 100 μl of bacteriocin buffer (0.1 M Tris [pH 7.5], 0.01 M DTT, and 0.5 M MgCl_2_). To determine bacteriocin antibiotic activity, 100 μg/10 μl of the CaroS1K protein solution was added to an indicator plate containing the Ea1068 strain growing on soft IFO-802 medium containing 0.65% agar. Growth inhibition zones at the point of addition were considered an indication of Carocin S1 activity (Fig. 3).

### Isolation of null alleles of the *flhD*, *fliC*, and *flhA *genes

Since flagella assembly requires the expression of both the *flhD *and *flhC *genes, we constructed the strain FlhD-KO (*flhD::Kan*). The linearized construct (containing the *flhD::Kan *DNA fragment) was transferred into H-rif-8-6, resulting in the homologous replacement of the native *flhD *gene and generating a null allele. The resultant kan and rif resistant transformants were screened by PCR with one set of primers (DY-SR1 and DY-SF1) representing the 5' and the 3' termini of the *flhD/C *operon. This set of primers generated a 1.3-kb product, if the transforming DNA was not integrated. However, a homologous replacement of the native *flhD *gene by the null allele yielded a 2.7-kb product. The observed PCR product was 2.7 kb, indicating that the *flhD *gene had been replaced by the null allele. The gene was therefore designated as *ΔflhD *(strain KH17).

To confirm that Carocin S1 was actually secreted via T3bSS, we selected two components of T3bSS for deletion analysis, the *fliC *and *flhA *genes. The *fliC *gene encodes a FliC protein, which is an outer membrane component of T3bSS. The linearized construct (containing the *fliC::Kan *DNA fragment) was transferred into H-rif-8-6, resulting in the homologous replacement of the native *fliC *gene and generating a null allele. The kan and rif resistant transformants were screened by PCR with one set of primers (fliC-sen and fliC-anti) representing the 5' and the 3' termini of the *fliC *operon. The gene was therefore designated as *ΔfliC *(strain FliC-KO). The flagellin-associated gene *flhA *encodes the inner membrane FlhA component of T3bSS. The same procedure was used to obtain the *flhA *knockout (KO) mutant, and the gene was designated *ΔflhA *(strain FlhA-KO).

### Complementation and analysis of *flhD*, *flhC*, *fliC*, and *flhA *genes

Wild-type H-rif-8-6 was used as a control and transformed with plasmids containing the *flhD *(pBYL2D) and *flhC *(pBYL2C) genes as well as the *flhD/C *(pBYL2DC) operon. The effect of these transformations on the bacteriocin production and cell size of the wild-type strain was assessed. The mutations in the *flhC *gene (TH12-2::Tn*5*) and the *ΔflhD *(KH17) gene were complemented by introduction of the *flhD*^+^(pBYL2D), *flhC*+(pBYL2C), and *flhD/C *(pBYL2DC) genes, and the effects of these respective genes were evaluated (see below).

**Figure 4:**
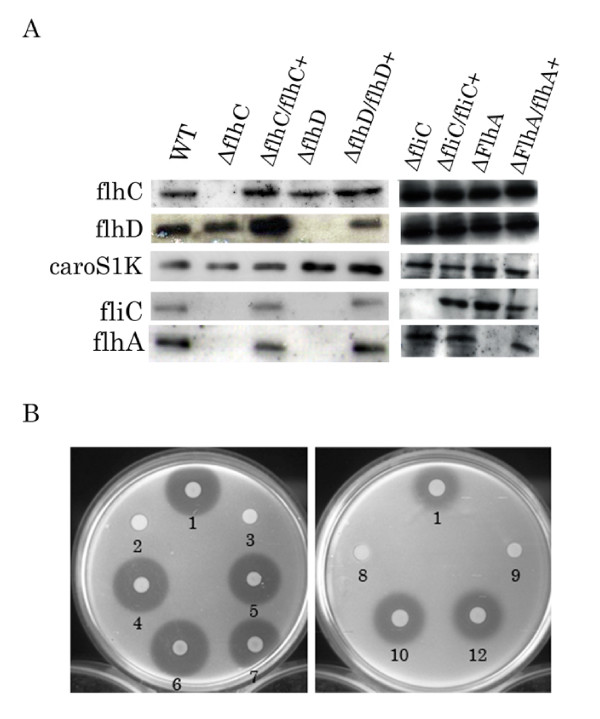
**Bacteriocin activity and transcription analysis of *Pectobacterium carotovorum *subsp. *carotovorum***. ***(A) Transcription analysis of the flhD*, *flhC*, *caroS1K*, and *fliC genes***. Total RNA (20 μg) from H-rif-8-6 (WT), TH12-2 (Δ*flhC*), TH12-2/pBYL2C (Δ*flhC*/*flhC+*), KH17 (Δ*flhD*), KH17/pBYL2D (Δ*flhD/flhD+*), FliC-KO (Δ*fliC*), FliC-KO/pBFC (Δ*fliC/fliC+*), FlhA-KO (Δ*flhA*), and FlhA-KO/pBFA (Δ*flhA/flhA+*) cells incubated in BSM medium at 28°C for 24 h was subjected to Northern blot analysis. Strain Ea1068 was used as an indicator for bacteriocin activity. ***(B) Bacteriocin activity assay***. Numbered strains: 1, H-rif-8-6 (wild type); 2, TH12-2 (Δ*flhC*); 3, KH17 (Δ*flhD*); 4, TH12-2/pBYL2C (Δ*flhC*/*flhC+*); 5, TH12-2/pBYL2DC (Δ*flhC*/*flhDC+*); 6, KH17/pBYL2D (Δ*flhD*/*flhD+*); 7, KH17/pBYL2DC (Δ*flhD*/*flhDC+*); 8, FliC-KO (Δ*fliC*); 9, FlhA-KO (Δ*flhA*); 10, FliC-KO/pBFC (Δ*fliC/fliC+*); and 11, FlhA-KO/pBFA (Δ*flhA/flhA+*). Strain Ea1068 was used as an indicator for bacteriocin activity.

Plasmids pBYL2DC (containing *flhD/C *gene), pBYL2C (containing the *flhC *gene), pBYL2D (containing the *flhD *gene), pBFC (containing the *fliC *gene), and pBFA (containing the *flhA *gene) were expressed from their own native promoters in *Pectobacterium carotovorum *subsp. *carotovorum *TH12-2, KH17, FliC-KO, and FlhA-KO strains. Northern blot analysis of total RNA from H-rif-8-6, TH12-2, TH12-2/pBYL2C, KH17, KH17/pBYL2D, FliC-KO/pBFC, and FlhA-KO/pBFA cells incubated in BSM medium at 28°C for 24 h. PCR products specific for *flhD*, *flhC*, *fliC*, *flhA*, and *caroS1K *were used as probes in the hybridizations. The data indicate the presence of *caroS1K *gene in all strains and the presence of the other genes in all strains except the uncomplemented mutants, as expected (Fig. 4A).

### Bacteriocin activity assay for complementation

Assay of bacteriocin secreted from the insertion mutants, with and without complementation, indicated that, after complementation, mutants recovered the ability to secrete LMWB. Their larger inhibition zones were comparable in diameter to those of their parent strain, H-rif-8-6 (Fig. 4B). Neither the KH17 nor TH12-2 strains could secrete Carocin S1. However, complementation (by transformation of KH17 and TH12-2 with the *flhD *and *flhC *genes), respectively, rescued the ability of these strains to secrete Carocin S1 and thereby increased inhibition zone diameters, which were comparable in size to that of wild type. After transformation, all deletion strains harboring their respective complementing plasmids secreted LMWB (Fig. 4B).

### Motility

**Figure 5:**
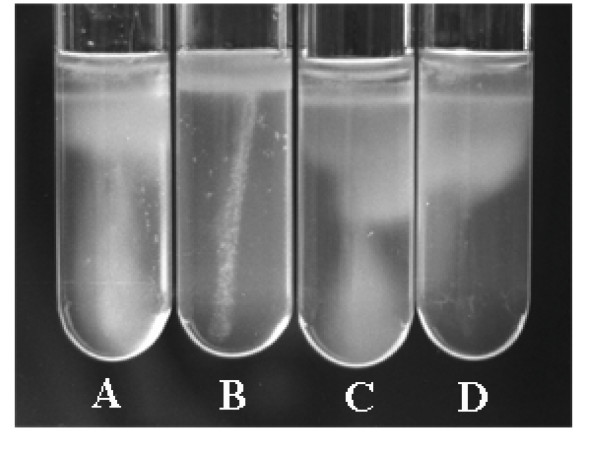
**Assay of motility in IFO-802 medium containing 0.5% agar, incubated at 25°C, over one month**. A: H-rif-8-6 (parent), B: TH12-2 (Δ*flhC*), C: TH12-2/pBYL2DC (Δ*flhC*/*flhDC+*), and D: TH12-2/pBYL2C (Δ*flhC*/*flhC+*).

The wild-type strain, H-rif-8-6, but not the transposon insertion mutant, TH12-2, was motile (Fig. 5). The motility of TH12-2 was restored by transformation with the *flhC *(pBYL2C) and *flhD/C *(pBYL2DC) genes.

## Discussion

In this study, the sequences of the *flhD *and *flhC *genes from *Pectobacterium carotovorum *subsp. *carotovorum *were highly homologous to the reported sequences of *flhD/C *genes in other bacterial strains [9-11,29,30]. These genes are adjacent and appear to share the same promoter [11]. Cloning of the *flhD/C *gene and subsequent transfer into the insertion mutant TH12-2 (*flhC::Tn5*) resulted in the recovery of bacteriocin activity (secretion of Carocin S1) in this mutant. The homologous replacement of the *flhD *gene by its null allele also resulted in the inhibition of Carocin S1 production. This indicated that both *flhD *and *flhC *are required for the production of Carocin S1 and, therefore, that the entire *flhD/C *operon influences the production of Carocin S1. FlhD has been previously shown to be associated with other stress-response systems [29,31]. Interestingly, flagella formation is controlled by the *flhD/C *operon [29]. In Gram-negative bacteria, the flagellar system is also known as the type III bacterium-flagella secretion system. Expression of the *flhD/C *genes is a form of response to environmental stress and requires the heat shock proteins DnaK, DnaJ, and GrpE [23], which are all related to environmental stress. Furthermore, the microcin B12 (*mcbA*) promoter is positively regulated by *flhD *[32,33]. It is therefore entirely appropriate to suggest that Carocin S1, which is normally induced by stress inducers like UV light and high competition from other related bacterial strains, is also under the control of *flhD/C*.

Although *flhD/C *was shown to control extracellular protein production through cumulative effects on *hexA *and *gacA *expression, this result was only demonstrated at the level of RNA transcription [34]. In this study, both the *flhC *and *flhD *genes regulated Carocin S1 secretion but not the transcription of the LMWB mRNA, *caroS1K*. Furthermore, assay of bacteriocin activity from TH12-2 (*ΔflhC*) detected intracellular but not extracellular Carocin S1 protein (Fig. 3). Similarly, we also found the transposon Tn*5 *insertion mutant, TH12-2 (Δ*fliC*), lost the ability to produce LMWB (data no shown). Northern blot analysis to monitor the expression of the *caroS1K *and *fliC *genes in the TH12-2 and KH17 strains detected the expression of *caroS1K *mRNA but not expression of *fliC *mRNA (Fig. 4A). However, as mentioned above, *flhD/C *genes regulate Gram-negative flagella synthesis and cell motility.

These results suggest that the *flhD/C *genes regulate the synthesis of bacterial flagella, which function as a flagellar type III secretion system (T3bSS) in Gram-negative bacteria, and that Carocin S1 utilizes this secretion machinery in *Pectobacterium carotovorum *subsp. *carotovorum*. However, because the growth of TH12-2 (*fliC*::Tn*5*) was extremely poor, this strain was lost before further experiments could be conducted. To further support our hypothesis, homologous replacement of the *fliC *gene (an outer membrane component of T3bSS) and the *flhA *gene (an inner membrane component of T3bSS) was used to generate a *fliC *null allele and *flhA *null allele, respectively. Bacteriocin analysis of extracellular fluids from the FliC-KO (*fliC::kan*) and FlhA-KO (*flhA::Kan*) strains also indicated significant inhibition of LMWB secretion. These results were similar to those found for TH12-2. Importantly, all these mutants still expressed the *caroS1K *mRNA.

The above results suggest a new function for the type III secretory system in this bacterial strain. Interestingly, complementation analysis of the *fliC *and *flhA *genes sometimes produced a smaller bacteriocin inhibition zone (3–8 mm *versus *8 mm for the wild type). These results indicated that although the *fliC *and *flhA *genes are expressed in the FliC-KO/pBFC and FlhA-KO/pBFA strains, the secretion of the CaroS1K protein is not as efficient as in the wild-type strain, H-rif-8-6. In this study, the *fliC *and *flhA *genes were inserted into FliC-KO and FlhA-KO cells using multicopy plasmids for overexpression. It is therefore possible that the FliC or FlhA protein is not efficiently recruited into the T3bSS, and consequently CaroS1K cannot be secreted competently. Interestingly, the results of *flhG *[16] and *fliC *[15] gene complementation are similar to those found in our studies. These studies also support our hypothesis.

In previous studies, just one mechanism was utilized by Gram-negative plant and animal pathogens for T3bSS secretion and translocation of virulence determinants into susceptible eukaryotic cells [17]. The present study uniquely demonstrates that *Pectobacterium *cells can transfer Carocin S1 extracellularly using the T3bSS system and kill related bacterial cells.

The observed smaller size of *flhD *mutant cells confirms the observation of Prüss and Matsumura [35-39] and corroborates the suggestion that *flhD *is responsible for cell elongation. Interestingly, TH12-2 (*flhC::Tn5*) cells are longer (our unpublished data), which indicates that *flhC *also controls cell elongation. This is similar to what was observed in *brg *insertion mutants [6], indicating a possible interference with or disruption of cell division. This is directly opposite to what was observed in *flhD *mutants. It could therefore be proposed that though *flhD *inhibits cell division [31,35], *flhC *may promote cell division in this bacterial strain. Therefore, the *flhC *gene may have functions unrelated to its role in the flagellar regulon, which may be opposite to that of *flhD*. However, both *flhD *and *flhC *are required for determining bacterial cell size.

## Conclusion

Based on these results, we conclude that the extracellular export of LMWB, Carocin S1, by *Pectobacterium carotovorum *subsp. *carotovorum *utilizes the type III secretion system, which also controls this bacterium's cell motility and cell size.

## Authors' contributions

YC participated in the bacteriocin analysis and construction of the null alleles of the *fliC *and *flhA *genes. DC conceived the study, participated in its design, and corrected the manuscript. All authors read and approved the final manuscript.
